# From digital companionship to capability building: reframing conversational artificial intelligence and older adults' psychological wellbeing in active aging

**DOI:** 10.3389/fpsyg.2026.1915597

**Published:** 2026-09-22

**Authors:** Chong Zhou, Hongyu Wu, Gaohang Li, Yuanxin Chen

**Affiliations:** 1 Economy & Management School, Wuhan Sports University, Wuhan, China; 2 Physical Education and Sports College, Central China Normal University, Wuhan, China

**Keywords:** active aging, conversational artificial intelligence, digital companionship, human-AI interaction, older adults, psychological wellbeing

## Abstract

The rapid diffusion of conversational artificial intelligence (AI) has reshaped the landscape of digital interaction in later life, with growing attention to its implications for older adults' mental health and wellbeing. Existing research has predominantly interpreted conversational AI through the lens of digital companionship, emphasizing its capacity to simulate social presence, reduce loneliness, and provide emotional support. While this perspective captures important affective outcomes, it offers a limited account of the developmental processes through which AI engagement may influence psychological wellbeing in aging populations. This conceptual analysis proposes a shift from a companionship-oriented paradigm to a capability-building perspective grounded in the Capability Approach. It argues that the primary value of conversational AI lies not in its ability to substitute social interaction, but in its potential to expand older adults' cognitive, emotional, social, and digital capabilities. Building on this argument, this paper develops an AI-Augmented Active Aging Framework that explains how conversational AI affordances, responsiveness, personalization, accessibility, and availability, interact with personal, social, environmental, and system-level conversion factors. Under supportive conditions, these affordances may facilitate cognitive, emotional, social, and digital capability formation, thereby supporting autonomy, competence, social integration, and active aging. Under adverse conditions, however, conversational AI may contribute to capability erosion through over-reliance, cognitive offloading, reduced human contact, or diminished autonomous judgment. The framework therefore emphasizes heterogeneous and conditional outcomes rather than assuming uniform benefits across older adults.

## 1 Introduction

Population aging has become one of the most consequential demographic transformations of the twenty-first century. The proportion of the global population aged 60 years and older is projected to increase from 12% in 2015 to 22% in 2050. In absolute terms, the number of people in this age group is expected to reach approximately 2.1 billion by 2050 (WHO, 2021). This transition has shifted scholarly and policy attention from longevity itself to the conditions under which later life can be experienced with autonomy, participation, psychological wellbeing, and meaningful engagement. The WHO's healthy aging framework defines aging well as the process of developing and maintaining functional ability that enables wellbeing in older age, placing the interaction between individual capacities and environmental conditions at the center of later-life development (Beard et al., 2016).

Psychological wellbeing is central to this framework. Earlier biomedical approaches often defined successful aging through the absence of disease or disability, but such definitions have been criticized for excluding older adults who live meaningful lives despite chronic conditions or functional limitations (Bowling and Dieppe, 2005). Contemporary aging scholarship gives greater attention to autonomy, purpose, social participation, resilience, and perceived quality of life. Older adults constitute a heterogeneous population whose experiences of health, autonomy, social connection, and psychological wellbeing vary considerably. Many older adults maintain strong interpersonal networks and participate actively in family, community, cultural, and digital life. Loneliness and social isolation nevertheless affect particular groups, especially under conditions such as bereavement, mobility restriction, institutionalization, poor health, or limited social support. Social isolation refers to the objective absence or scarcity of social contacts, whereas loneliness reflects a subjective discrepancy between desired and actual relationships (Cornwell and Waite, 2009). When present, both are associated with adverse health outcomes, including increased mortality risk (Holt-Lunstad et al., 2015).

The digital divide among older adults is shaped not only by access to devices or connectivity, but also by skills, motivation, confidence, usage patterns, and the ability to convert digital use into meaningful outcomes (van Deursen and Helsper, 2015; Van Dijk, 2020). Studies of older adults' digital engagement further show that age-related digital inequalities are shaped by education, everyday ICT practices, perceived usefulness, and confidence in technology use (Friemel, 2016; Quan-Haase et al., 2016). Digital exclusion may overlap with social exclusion, creating a double burden for individuals already vulnerable to isolation (Seifert et al., 2021).

Conversational artificial intelligence represents a distinctive development within this digital landscape. Unlike earlier digital tools that depend on menus, commands, or complex interface navigation, conversational AI enables interaction through natural language. Advances in large language models have enhanced the capacity of conversational systems to generate fluent, context-sensitive, and personalized dialogue. Recent studies focusing on older adults further highlight the importance of accessibility, trust, user control, and context-sensitive design in translating these technological capacities into meaningful support (Dwivedi et al., 2023; Feuerriegel et al., 2024; Huang et al., 2025; Zheng et al., 2026). This interactional form is relevant for older adults because it may lower procedural barriers associated with traditional digital interfaces and allow users to express needs, ask questions, clarify meanings, and receive feedback in ways that resemble everyday communication.

Recent advances in generative AI, multimodal models, and adaptive AI systems have expanded the functional boundaries of conversational technologies. Contemporary AI systems increasingly integrate contextual information, diverse data modalities, and adaptive interaction mechanisms, which may reshape how older adults access information, regulate emotions, participate socially, and navigate digital environments. However, these technological advances also introduce new uncertainties regarding reliability, transparency, privacy, and human autonomy. Therefore, the relationship between conversational AI and later-life wellbeing requires a framework that considers not only expanded technological affordances but also the conditions through which these affordances are converted into meaningful capabilities.

Conversational AI has also become increasingly important in mental health and wellbeing research. Reviews of mental health chatbots indicate that conversational agents have been used for psychoeducation, mood tracking, emotional support, cognitive behavioral techniques, and self-management support (Abd-alrazaq et al., 2019). Evidence from randomized trials suggests that fully automated conversational agents can reduce symptoms of depression and anxiety under certain conditions (Fitzpatrick et al., 2017). Recent systematic reviews further suggest that AI-based conversational agents may promote mental health and wellbeing, although the mechanisms through which such effects occur remain insufficiently specified (Li et al., 2023).

Within a substantial part of aging and care-technology research, conversational AI has been interpreted through the lens of digital companionship. This orientation is partly rooted in earlier studies of socially assistive robots and relational agents, which examined whether artificial agents could provide social presence, emotional reassurance, and psychosocial support for older adults experiencing loneliness or reduced interpersonal contact (Bemelmans et al., 2012; Pu et al., 2019; Wada and Shibata, 2007). This body of research should not be read as evidence that all older adults are lonely or seek artificial companionship. Rather, digital companionship represents one influential framework through which socially interactive technologies have been evaluated, particularly in care and mental health contexts.

However, companionship-oriented explanations remain theoretically limited. They tend to evaluate conversational AI primarily in terms of emotional support, perceived social presence, and loneliness reduction. These outcomes matter, but they do not fully capture the developmental processes through which AI interaction may influence psychological wellbeing. Psychological wellbeing involves more than positive affect or reduced loneliness. It includes autonomy, environmental mastery, personal growth, purpose in life, and meaningful social connection (Keyes, 2002; Ryff, 1989). A companionship framework can explain how AI may provide comfort, but it is less able to explain how AI may support learning, emotional regulation, decision-making, digital participation, and adaptive functioning.

The capability approach provides a theoretical foundation for this broader interpretation. Sen conceptualizes wellbeing in terms of substantive freedoms to achieve valued functionings, shifting attention from resources themselves to what individuals are able to do and be (Sen, 1999). Nussbaum further emphasizes that human development requires opportunities for practical reason, affiliation, learning, and control over one's environment (Nussbaum, 2011). Applied to conversational AI, this perspective suggests that the key question is not whether AI provides companionship, but whether AI interaction expands the capabilities through which older adults pursue valued forms of living.

This paper therefore argues that conversational AI should be understood not primarily as a source of digital companionship, but as a capability-building resource. It proposes that conversational AI may contribute to older adults' psychological wellbeing by supporting the development of cognitive, emotional, social, and digital capabilities. Building on this argument, this paper develops an AI-Augmented Active Aging Framework that explains how conversational AI affordances can be translated into capability formation, psychological wellbeing, and active aging outcomes. This conceptual analysis was informed by literature across conversational AI, aging and wellbeing, human–AI interaction, digital inclusion, and the Capability Approach. Relevant studies were identified from major academic databases based on their theoretical relevance and empirical contribution to the development of the proposed framework. The purpose was conceptual integration rather than exhaustive evidence synthesis.

## 2 Beyond digital companionship: reconsidering the dominant paradigm

Conversational AI and related socially interactive technologies have frequently been interpreted through the lens of digital companionship in aging research. This orientation has emphasized emotional reassurance, perceived social presence, and interactional continuity, particularly in contexts involving reduced interpersonal contact or increased care needs. The digital companionship paradigm should be understood as an influential research framing rather than a universal description of older adults' needs. Older adults vary substantially in their social networks, health, education, socioeconomic resources, digital experience, life goals, and interest in conversational technologies. Many use digital systems for information seeking, learning, creativity, civic participation, and everyday problem-solving without seeking companionship. The concern addressed in this paper is therefore not that all older adults require artificial companionship, but that conversational AI has often been evaluated through a relatively narrow set of companionship-related outcomes.

Early studies of socially assistive robots provided an important empirical basis for this interpretation. Robotic companions such as PARO were introduced into eldercare settings to stimulate engagement, reduce distress, and create affective interaction among older adults. Studies of seal-like companion robots reported socio-psychological and physiological benefits in care environments, including improved mood and increased social interaction (Wada and Shibata, 2007). Subsequent reviews found that socially assistive robots may improve engagement and reduce loneliness among older adults, although effects vary across settings, intervention duration, and user characteristics (Abdi et al., 2018; Bemelmans et al., 2012). Meta-analytic evidence further suggests that robot-assisted interventions can have positive effects on loneliness and quality of life, particularly in institutional care contexts (Pu et al., 2019). Broader reviews of healthcare robot acceptance also indicate that older adults' responses to robotic technologies are shaped by perceived usefulness, ease of use, trust, and the social meanings attached to care technologies (Broadbent et al., 2009). Experimental evidence from companion robot interventions further suggests that artificial companions may produce psychosocial benefits, although such effects remain sensitive to design, setting, and user characteristics (Robinson et al., 2013).

A related stream of work on relational agents and embodied conversational systems reinforced this companionship framing. Relational agents were designed to maintain long-term socio-emotional relationships with users rather than merely deliver information. In studies involving older adults, participants often described interaction with relational agents in interpersonal terms, sometimes comparing them to friendly conversational partners (Bickmore et al., 2005). Human-computer interaction research further shows that older adults' engagement with technology is often shaped by discourses of dependency, vulnerability, and care (Vines et al., 2015). When aging is framed primarily through isolation, care need, or technological dependency, artificial agents are easily positioned as compensatory companions.

Mental health chatbots extended this trajectory into psychological support. Systems such as Woebot demonstrated that fully automated conversational agents could deliver elements of cognitive behavioral support and reduce symptoms of depression and anxiety in non-clinical populations (Fitzpatrick et al., 2017). Other digital mental wellbeing systems, such as Wysa, emphasized empathetic dialogue, emotional support, and real-time availability (Inkster et al., 2018). Reviews of mental health chatbots show that many systems are designed around psychoeducation, self-monitoring, emotional expression, and supportive conversation (Vaidyam et al., 2019). Although these systems are not designed solely as companions, their conversational form encourages interpretation through relational and supportive categories.

Recent large language model-based systems have made this interpretation more salient. Unlike earlier rule-based chatbots, contemporary conversational AI can sustain open-ended dialogue, generate context-sensitive responses, and adapt communicative style across diverse topics. However, recent evaluations indicate that conversational fluency does not necessarily guarantee reliability, factual accuracy, or appropriate support, particularly in health-related contexts (Ji et al., 2023; Langston et al., 2024; Stade et al., 2024). These capacities intensify perceptions of social presence and make it easier for users to experience AI interaction as conversationally meaningful. Generative AI therefore strengthens the psychological plausibility of digital companionship, particularly for older adults who may use conversational systems for daily communication, emotional expression, information seeking, or reassurance (Dwivedi et al., 2023).

Despite their diversity, these research strands converge on a shared interpretive frame: conversational AI is primarily evaluated according to its capacity to simulate companionship, provide emotional support, and reduce loneliness. This framing has generated valuable insights into the affective and relational dimensions of human-AI interaction. However, its explanatory scope remains limited because it tends to concentrate on affective outcomes while giving less attention to developmental processes underlying psychological wellbeing.

This limitation becomes clear when conversational AI is assessed against broader theories of wellbeing. Psychological wellbeing extends beyond emotional comfort or loneliness reduction. It includes autonomy, environmental mastery, personal growth, purpose in life, positive relations, and self-acceptance (Ryff, 1989). Keyes similarly distinguishes flourishing from the mere absence of distress (Keyes, 2002). A conversational AI system may reduce loneliness without necessarily enhancing agency, competence, autonomy, or meaningful participation. When conversational AI is conceptualized primarily as a companion, older adults are positioned mainly as recipients of affective support rather than as agents of capability development.

A second limitation concerns temporal depth. Companionship-oriented studies often examine immediate or short-term outcomes, such as mood, satisfaction, perceived social presence, or post-intervention loneliness. These outcomes matter, but they provide limited insight into aging as an adaptive process. Baltes' theory of selection, optimization, and compensation emphasizes that later-life development depends on the ongoing management of changing resources, goals, and constraints (Baltes, 1997). Conversational AI may contribute to such adaptation through repeated learning, reflective practice, decision support, and digital skill formation, yet these processes are not easily captured by companionship-based indicators.

A third limitation arises from the multifunctionality of contemporary conversational AI. Current systems are not limited to emotional conversation. They can help users interpret health information, compare options, organize routines, practice communication, and learn digital procedures. Healthcare research on conversational agents shows that these systems increasingly perform informational, motivational, and self-management functions (Kocaballi et al., 2019; Laranjo et al., 2018). Mental health research also indicates that chatbot interaction may involve self-disclosure, cognitive reframing, and emotional processing rather than simple companionship dynamics (Ho et al., 2018). When these functions are interpreted only through companionship, the broader developmental potential of conversational AI remains under-theorized.

Human-AI interaction research also cautions against overextending relational metaphors. Users often bring social expectations to conversational agents, yet these expectations can exceed system capabilities. The mismatch between anticipated conversational competence and actual interaction quality has been described as a gulf between user expectation and experience (Luger and Sellen, 2016). This issue is especially important in mental health and aging contexts, where vulnerable users may attribute excessive understanding, reliability, or care capacity to systems that remain limited, opaque, and error-prone. Critical AI scholarship further shows that large language models may generate fluent but unreliable outputs, reproduce biases, and obscure the social and material conditions under which AI systems are produced (Bender et al., 2021; Crawford, 2021). Ethical debates around care technologies similarly caution that simulated social presence can blur boundaries between support, substitution, and social neglect (Sharkey and Sharkey, 2012).

These developments indicate that the digital companionship paradigm captures only a partial dimension of conversational AI in later life. It explains emotional comfort, perceived presence, and loneliness reduction, but it provides limited analytical leverage for understanding learning, reflection, agency, participation, and adaptive functioning. This limitation does not require rejecting companionship as a concept. Rather, it requires repositioning companionship as one narrower pathway within a broader developmental account of conversational AI.

Figure 1 synthesizes this transition. It shows how companionship-related effects are retained within a broader capability-building perspective while recognizing heterogeneity in older adults' goals, existing strengths, social resources, health conditions, and digital experience.

**Figure 1:**
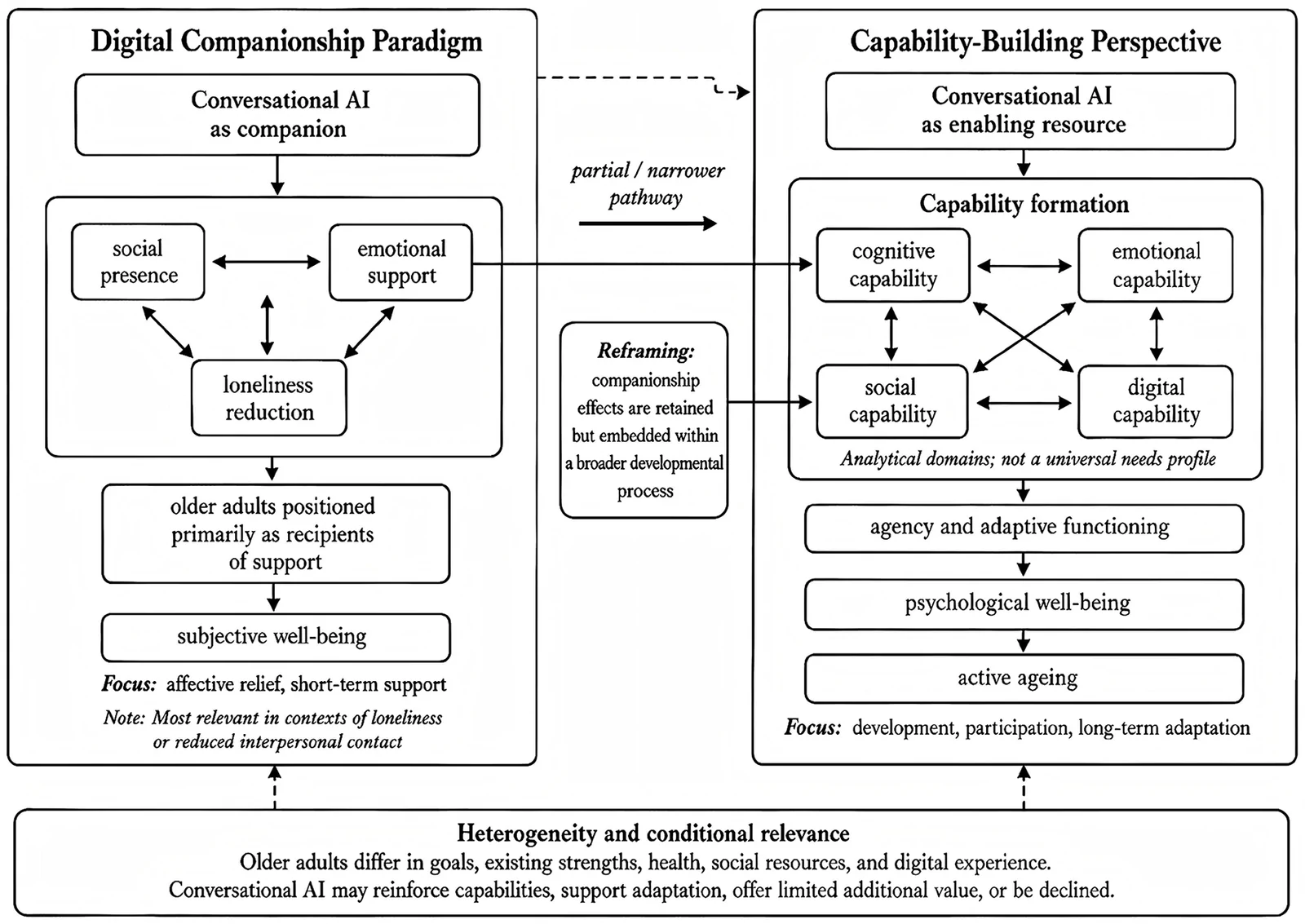
From digital companionship to capability building in conversational ai and older adults' wellbeing. Note. The capability-building perspective subsumes and extends the digital companionship paradigm by embedding companionship-related effects within a broader developmental process. The relevance and outcomes of both pathways vary according to older adults' goals, existing strengths, health, social resources, and digital experience.

A broader conceptualization is therefore required. Conversational AI should be examined not only in terms of whether it provides companionship, but also in terms of whether it expands older adults' capacity to pursue valued forms of living. The central analytical question shifts from whether AI can alleviate loneliness to whether AI interaction can support cognitive, emotional, social, and digital capabilities that enable autonomy, participation, and adaptation in later life. The next section develops this capability-building perspective.

## 3 Conversational AI as a capability-building resource

The limitations of the digital companionship paradigm suggest the need for a broader framework capable of explaining how conversational AI contributes to psychological wellbeing beyond emotional support. Companionship-oriented approaches focus on what conversational AI provides, whereas a capability perspective asks what older adults become able to do through sustained interaction with such systems. This distinction is fundamental because wellbeing depends not only on supportive resources but also on individuals' ability to transform those resources into meaningful forms of action, participation, and self-development. A capability-building perspective does not begin from the assumption that older adults are uniformly deficient in cognitive, emotional, social, or digital functioning. Capability refers to the substantive opportunities available to individuals to pursue valued activities, and these opportunities differ across people and contexts. Conversational AI may reinforce existing strengths, support adaptation to specific transitions, expand access to new activities, or offer limited additional value for users whose goals and resources do not require such support.

The capability approach offers a theoretical foundation for this shift. Developed by Sen and elaborated by Nussbaum, it critiques welfare perspectives that equate wellbeing with resource possession or subjective satisfaction. Capabilities refer to the substantive freedoms individuals possess to achieve valued functionings, while functionings are the realized states of being and doing that constitute a person's life (Robeyns, 2005). From this perspective, resources are valuable only insofar as they contribute to capability formation. The same resource may generate different outcomes depending on personal characteristics, social environments, and institutional conditions.

This insight is particularly relevant in later life, where health conditions, mobility limitations, social transitions, and technological change reshape the opportunities available to older adults. Individuals vary considerably in their ability to adapt to these changes and transform available resources into meaningful outcomes. Consequently, understanding wellbeing in later life requires attention to capability rather than resources alone.

### 3.1 Conversational AI as a conversion resource

Conversational AI can be understood as a conversion resource that helps individuals transform available technological opportunities into valued functionings. Its significance lies not in the direct benefits it provides, but in its capacity to facilitate capability development (Alkire, 2005). Through natural language interaction, older adults can acquire knowledge, explore alternatives, solve problems, and receive guidance in ways that would otherwise require substantial digital skills or external support.

This interpretation marks a departure from the companionship paradigm. Rather than viewing AI primarily as a source of emotional comfort, the capability perspective interprets AI as an enabling infrastructure that expands opportunities for action. Conversational AI differs from earlier digital technologies because it reduces demands associated with search procedures, navigation structures, and technical commands. Information seeking, learning, and problem solving become more accessible to individuals who might otherwise face technological barriers.

### 3.2 Cognitive capability

Cognitive capability refers to the ability to acquire information, interpret knowledge, solve problems, and make informed decisions. Cognitive functioning plays a critical role in maintaining autonomy and quality of life throughout later adulthood. Computer-based cognitive interventions can support cognitive engagement and training among healthy older adults and older adults with mild cognitive impairment, although outcomes vary across intervention designs and measures (Alnajjar et al., 2019). Conversational AI extends this potential by transforming information retrieval into an interactive learning process.

Unlike conventional search engines, conversational systems allow users to ask follow-up questions, request clarification, compare alternatives, and receive explanations adapted to their level of understanding. These interactions are especially relevant when older adults face decisions concerning health management, financial planning, social participation, or technology use. From a capability perspective, the outcome is not simply knowing more information but becoming more capable of reasoning, learning, and making informed choices.

### 3.3 Emotional capability

Emotional capability refers to the capacity to recognize, interpret, express, and regulate emotions in ways that support psychological adaptation. Emotion regulation theory emphasizes that emotional wellbeing depends not only on external support but also on individuals' ability to manage emotional experiences through cognitive and behavioral processes (Gross, 2015). Conversational AI may contribute to this capability by creating opportunities for emotional articulation, reflection, and cognitive reappraisal.

The distinction between emotional support and emotional capability lies in the durability and transferability of the outcome. Emotional support refers to comfort, reassurance, or reduced distress that remains contingent on the interaction itself. Emotional capability is present when conversational engagement strengthens a user's ability to identify emotions, select regulation strategies, reinterpret difficult experiences, and communicate emotional needs across situations, including when the system is no longer present. Articulation, reflection, and low-risk disclosure may contribute to either outcome; they constitute capability building only when they produce persistent and transferable self-regulatory competence.

This distinction can be examined empirically. Companionship-related support may be indicated by immediate changes in mood, perceived empathy, or loneliness following interaction. Emotional capability requires evidence of change in emotion-regulation self-efficacy, independent strategy use, reflective competence, or coping across time and contexts. A system that repeatedly calms a user without improving independent regulation provides support but does not necessarily build emotional capability. Recent research on large language models in mental health similarly emphasizes that conversational responsiveness should not be equated with psychological benefit, and that emotional outcomes require careful validation through multidimensional assessment (Hua et al., 2025; Stade et al., 2024).

### 3.4 Social capability

Social capability refers to the ability to establish, maintain, and navigate social relationships and community engagement. Social participation remains one of the strongest determinants of health and wellbeing in later life (Walker, 2002). The companionship paradigm often assumes that conversational AI contributes to social wellbeing by replacing missing interaction. The capability perspective proposes a different mechanism: conversational AI may strengthen the capacities required for meaningful participation in social life.

Older adults may use conversational systems to prepare for conversations, obtain information about community opportunities, seek advice regarding interpersonal situations, or practice communication strategies. These activities may increase confidence and reduce barriers to engagement. This distinction matters because active aging emphasizes participation rather than passive connectedness (Walker, 2002). Social capability therefore offers a more development-oriented explanation of how conversational AI may influence social wellbeing.

### 3.5 Digital capability

Digital capability refers to the knowledge, skills, confidence, and autonomy required to engage effectively in digital environments. Access to services, information, healthcare, education, and social participation increasingly depends on digital infrastructures. Digital inequality is shaped not only by technological access but also by skills and patterns of use (Van Dijk, 2020). Many older adults continue to encounter barriers associated with interface complexity, technological anxiety, and limited digital experience (Hunsaker and Hargittai, 2018).

Conversational AI may reduce some of these barriers because natural language interaction lowers procedural demands. Users can interact through familiar communicative practices rather than specialized technical skills. Digital capability occupies a distinctive position because it also supports the development of other capabilities: it facilitates access to learning resources, communication opportunities, health information, and civic participation.

### 3.6 From companionship to capability

The four capability domains are analytical categories rather than a universal needs profile for older adults. Their relevance varies according to individual goals, existing strengths, life circumstances, and environmental demands. A socially active and digitally experienced older adult may use conversational AI primarily for learning or decision support, whereas another user may value assistance with digital service navigation or emotional reflection. Some older adults may derive no meaningful capability benefit or may choose not to use conversational AI. These domains clarify the distinction between companionship-oriented and capability-oriented interpretations of conversational AI. The companionship paradigm focuses on what individuals receive; the capability perspective focuses on what individuals become able to do. Companionship emphasizes emotional support and immediate relief; capability emphasizes human development, agency, participation, and adaptation.

This distinction aligns with empowerment theory, which conceptualizes empowerment as a process through which individuals gain greater control over decisions and actions affecting their lives (Zimmerman, 2000). Conversational AI contributes to empowerment when it strengthens individuals' capacity to learn, reflect, communicate, and participate. Under such conditions, AI functions not as a substitute for human agency but as a resource that supports its expansion. This reconceptualization establishes the theoretical foundation for understanding conversational AI as a developmental resource.

## 4 An AI-augmented active aging framework

The preceding sections have argued that conversational AI should be conceptualized not primarily as a source of digital companionship but as a capability-building resource. This reconceptualization raises a central question: through what mechanisms does interaction with conversational AI contribute to older adults' psychological wellbeing and active aging?

Existing studies often assume a direct relationship between AI use and psychological outcomes. Conversational agents are expected to reduce loneliness, improve mood, or provide emotional support, and these effects are interpreted as indicators of wellbeing. Such explanations capture important outcomes but provide limited insight into the processes through which technological interaction produces developmental change.

To address this issue, this paper proposes an AI-Augmented Active Aging Framework. The framework integrates technological affordance theory, the Capability approach, and active aging theory. Its central proposition is that conversational AI contributes to psychological wellbeing indirectly through capability formation. AI does not improve wellbeing simply because users interact with it. Interaction creates opportunities for capability development, and capability development subsequently supports psychological flourishing and active aging.

The logic of the proposed framework is presented in Figure 2, which provides the overall architecture for the following analysis. It shows that the effects of conversational AI affordances depend on personal, social, environmental, and system-level conversion factors. These conditions may support capability formation or contribute to capability erosion, with corresponding consequences for psychological wellbeing, participation, and active aging. Later outcomes may, in turn, reinforce or erode subsequent capability trajectories.

**Figure 2:**
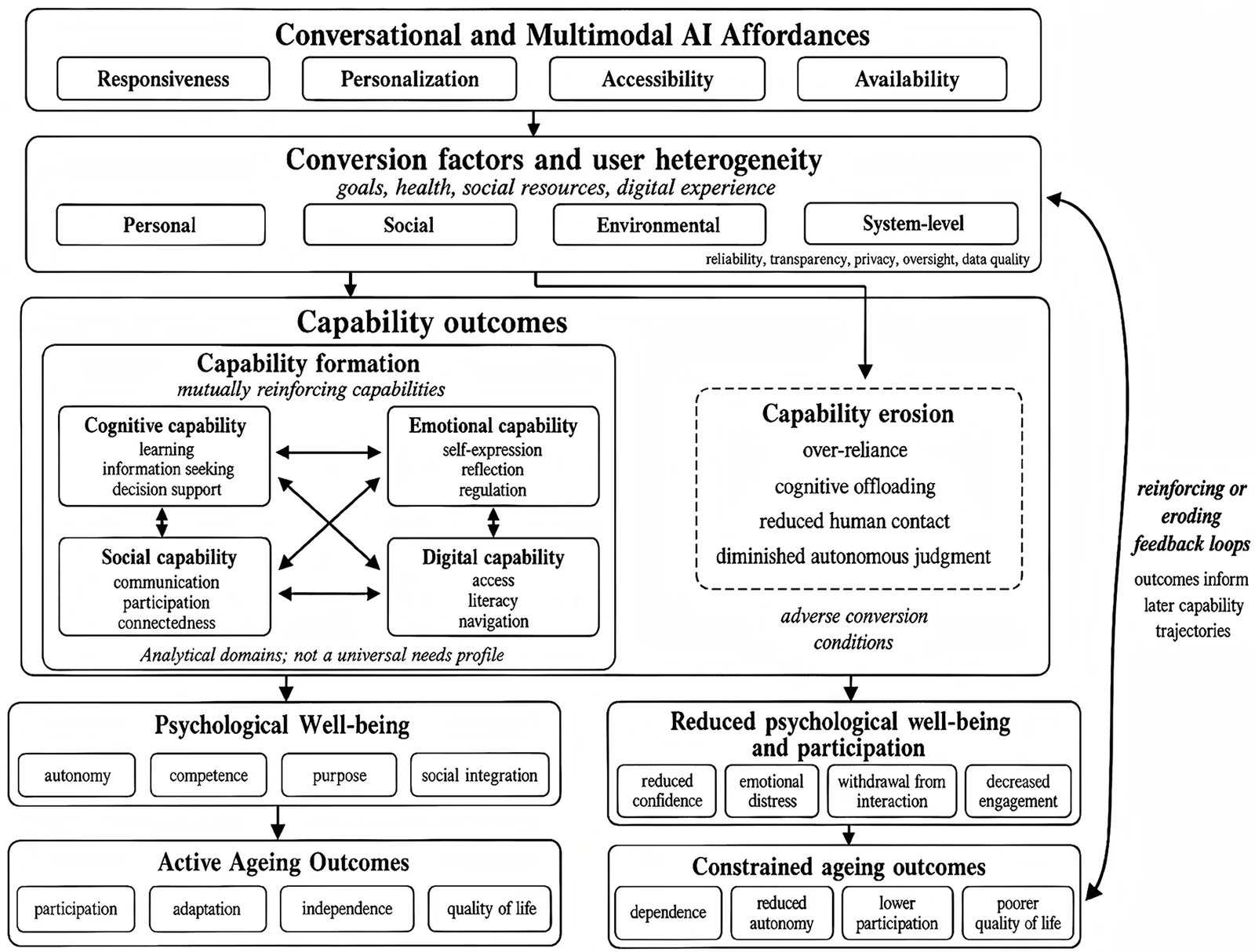
An AI-augmented capability framework for active aging with formation and erosion pathways. Note. Conversational and multimodal AI affordances operate through personal, social, environmental, and system-level conversion factors. Recent advances in adaptive and context-aware AI broaden these affordances but do not guarantee capability gains, which remain conditional on reliability, transparency, privacy, data quality, and human oversight.

### 4.1 Conversational AI affordances as developmental conditions

Affordances refer to the action possibilities that emerge through the interaction between individuals and technological environments rather than from technological features alone (Norman, 2013). This perspective is important because conversational AI differs from previous generations of digital technology. Traditional interfaces often require procedural knowledge, navigation skills, and technical competence, whereas conversational AI enables natural language interaction.

The four affordances included in the framework were selected according to three criteria. First, they describe interaction-level action possibilities that distinguish conversational AI from conventional menu-based or command-based digital interfaces. Second, they can plausibly support repeated processes of questioning, clarification, reflection, rehearsal, and guided task completion through which capability formation may occur. Third, they are sufficiently general to operate across cognitive, emotional, social, and digital domains rather than being tied to a single outcome. They therefore constitute a parsimonious set of developmental affordances rather than an exhaustive taxonomy of conversational AI characteristics. Other properties, including reliability, transparency, controllability, privacy protection, and anthropomorphic presentation, remain important but occupy a different position in the framework. Reliability, transparency, controllability, and privacy protection condition whether users can safely convert conversational interaction into valued capabilities. Anthropomorphism primarily shapes relational expectations and perceived social presence. These properties are therefore treated as conversion conditions or moderators rather than as the primary developmental affordances represented in the central pathway.

While recent generative and multimodal AI developments have expanded the technical capabilities of conversational systems, the framework focuses on interaction-level affordances through which such technologies may influence capability development. Four affordances are especially relevant to capability development among older adults. Responsiveness allows users to ask follow-up questions, seek clarification, and refine requests, transforming information retrieval into active engagement. Personalization allows systems to adapt explanations, communicative style, and recommendations to users' needs and circumstances, although its value depends on contextual understanding, system reliability, and user trust (Mathur et al., 2026). Accessibility lowers technological barriers by allowing users to focus on goals rather than procedures. Availability creates conditions for repeated engagement, which is essential because capability development is cumulative. Recent AI advances do not eliminate the importance of these affordances but extend their technological foundations. For example, personalization may increasingly incorporate contextual and multimodal information, while responsiveness and availability may be supported by more adaptive and continuous AI systems. However, these advances do not guarantee capability gains, as reliability, transparency, and human oversight remain essential conversion conditions. These affordances constitute enabling conditions rather than capability outcomes in themselves. Their effects overlap across capability domains and depend on personal, social, environmental, and system-level conversion factors.

### 4.2 Capability formation as the core mechanism

The framework positions capability formation as the primary mechanism linking conversational AI to psychological wellbeing. This proposition departs from companionship-based explanations, which typically assume that AI contributes directly to emotional outcomes. The capability perspective suggests that AI influences wellbeing indirectly by strengthening capacities that enable individuals to act, learn, adapt, and participate.

Through repeated interaction with increasingly adaptive AI systems, conversational AI may support cognitive capability through learning and decision-making, emotional capability through reflection and regulation, social capability through communication and participation; and digital capability through technological engagement. These capabilities form an interconnected system rather than independent outcomes. Digital capability may facilitate access to educational resources that enhance cognitive capability; improved cognitive capability may strengthen confidence and support social participation; social engagement may contribute to emotional wellbeing. Capability formation should therefore be understood as cumulative and mutually reinforcing.

Capability development is not equivalent to technology use. This distinction becomes increasingly important as AI systems become more autonomous and context-aware, because greater technological sophistication does not necessarily correspond to greater human agency. High levels of AI interaction do not automatically generate capability gains. Capabilities emerge only when individuals actively transform interactional opportunities into meaningful forms of learning, participation, and adaptation. This distinction reinforces a central principle of the capability approach: resources matter because of what individuals are able to do with them. Conversion may also generate adverse capability outcomes. Repeated use may weaken independent capability when conversational AI substitutes for individual reasoning, encourages habitual dependence, displaces meaningful human interaction, or provides unreliable guidance. Capability formation and capability erosion therefore represent alternative trajectories whose relative likelihood depends on users' goals, patterns of engagement, and personal, social, environmental, and system-level conversion conditions. These conditions may also produce unequal capability gains across groups of older adults. In Ambient Assisted Living contexts, conversational AI may also be combined with behavioral and physiological signals to provide complementary information for assessing capability trajectories, although such signals require validation against self-reports, behavioral observations, and contextual information rather than direct interpretation of psychological states (Liu et al., 2026).

While Figure 2 presents the overall mechanism, the framework also requires a more specific account of how different AI affordances relate to particular capability domains and wellbeing outcomes. Table 1 therefore translates the conceptual model into a set of analytically distinguishable pathways that can guide future empirical research.

**Table 1:** Capability-building pathways of conversational AI for older adults.

Capability domain	Illustrative AI affordances	Capability-building mechanism	Relevant conversion conditions	Psychological wellbeing pathway	Potential empirical indicators
Cognitive capability	Responsiveness; personalization; accessibility	Conversational AI supports iterative questioning, clarification, explanation, and decision support, enabling older adults to acquire knowledge, compare options, and organize information.	Baseline cognitive functioning; health and information literacy; ability to evaluate AI outputs; system reliability and transparency	Enhances environmental mastery and informed autonomy by strengthening users' ability to understand situations and make reasoned choices.	Perceived decision competence; information literacy; learning confidence; independent problem-solving; perceived environmental mastery
Emotional capability	Responsiveness; personalization; availability	Conversational AI provides opportunities for emotional articulation, reflective dialogue, cognitive reappraisal, and guided practice in emotion-regulation strategies.	Emotional readiness; privacy and perceived safety; access to human support; ability to transfer strategies beyond AI interaction	Supports self-regulation, resilience, and self-acceptance by strengthening users' capacity to interpret and manage emotional experiences independently.	Emotion-regulation self-efficacy; independent strategy use; transfer of coping skills; reflective competence; reduced reliance on AI reassurance
Social capability	Personalization; accessibility; responsiveness	Conversational AI supports communication rehearsal, interpersonal problem-solving, social confidence, and access to information about community participation.	Existing social networks; communication confidence; community opportunities; cultural norms; risk of displacement of human contact	Strengthens social integration and belonging by supporting users' capacity to initiate, maintain, and navigate social relationships.	Social confidence; communication self-efficacy; social participation frequency; perceived connectedness; belongingness
Digital capability	Accessibility; availability; responsiveness; personalization	Conversational AI lowers procedural barriers through natural-language interaction, step-by-step guidance, clarification, and repeated practice.	Device and connectivity access; sensory and interface accessibility; prior digital experience; literacy; affordability; language coverage	Promotes independence and digital inclusion by increasing users' confidence and autonomy in accessing digital services and resources.	Digital self-efficacy; service-navigation ability; technology-use confidence; independent task completion; digital participation

The pathways and affordance combinations are illustrative and non-exclusive. The same affordance may support multiple capability domains, and the same capability may be influenced by several affordances. These pathways do not imply that all older adults require support in each domain or that conversational AI produces equivalent outcomes across users. Capability effects depend on personal, social, environmental, and system-level conversion conditions.

### 4.3 Psychological wellbeing and active aging

Within the framework, capability formation may support psychological wellbeing, whereas capability erosion may contribute to reduced wellbeing and participation. Psychological wellbeing can be understood as positive functioning involving autonomy, competence, self-regulation, purpose, and social connection, rather than merely the reduction of distress or loneliness (Diener et al., 2010; Huppert, 2009). Cognitive capability supports informed decision-making and environmental mastery; emotional capability enhances resilience and self-regulation; social capability facilitates belonging and participation; digital capability expands access to opportunities and resources. Psychological wellbeing therefore reflects expanded functional possibilities rather than merely improved emotional states.

The final stage of the framework concerns active aging. Active aging is commonly defined as the process of optimizing opportunities for health, participation, and security in order to enhance quality of life as people age (Walker, 2002). Subsequent scholarship has further emphasized that active aging should be understood as a multidimensional and policy-relevant framework involving health, participation, autonomy, security, and life-course adaptation rather than a narrow measure of physical activity or labor-market activity (Fernández-Ballesteros et al., 2013; Foster and Walker, 2015). This broader interpretation also resonates with recent extensions of successful aging theory, which call for more inclusive accounts of adaptation, engagement, and heterogeneity in later life (Rowe and Kahn, 2015). Most active aging frameworks were developed before the emergence of contemporary conversational AI. The present framework addresses this gap by positioning conversational AI within the ecology of active aging resources. Capability expansion contributes to active aging through independent decision-making, adaptation to age-related transitions, community participation, digital inclusion, and access to services and information.

### 4.4 Dynamic feedback and theoretical propositions

The framework is intended to generate empirically vulnerable claims rather than to treat every null result as evidence of failed conversion. Its propositions apply under conditions of sustained and task-relevant engagement, basic user comprehension, sufficient system reliability, and the absence of severe access barriers. P1 would be challenged if these conditions were present but no measurable change occurred in any capability domain across repeated studies. P2 would be challenged if conversational AI engagement affected psychological wellbeing without corresponding and temporally prior capability change. P3 would be challenged if the indirect pathway through psychological wellbeing was consistently absent. P4 would be challenged if longitudinal or cross-lagged evidence consistently showed only unidirectional relationships. P5 would be challenged if adverse patterns of use and system conditions were not associated with capability erosion across repeated studies. P6 would be challenged if capability outcomes did not vary systematically according to users' goals and personal, social, environmental, or system-level conversion factors.

***P1*.** *Under conditions of sustained, task-relevant, comprehensible, and sufficiently reliable interaction, conversational AI and emerging multimodal AI affordances are associated with changes in older adults' cognitive, emotional, social, and digital capabilities*.

***P2*.** *Capability formation mediates the relationship between conversational AI engagement and psychological wellbeing*.

***P3*.** *Psychological wellbeing mediates the relationship between capability formation and active aging outcomes*.

***P4*.** *Capability formation, psychological wellbeing, active participation, and conversational AI engagement are reciprocally related over time*.

***P5*.** *Adverse conversion conditions may cause conversational AI use to contribute to capability erosion*.

***P6*.** *Unequal personal, social, environmental, and system-level conversion factors may produce divergent capability outcomes across groups of older adults*.

## 5 Theoretical contributions and future research agenda

The AI-augmented active aging framework extends current discussions of conversational AI, aging, and psychological wellbeing by shifting attention from emotional substitution to capability expansion. Existing research has generated valuable insights into conversational AI as a source of companionship, emotional support, and social presence. However, the dominance of companionship-oriented explanations has limited understanding of how AI interaction contributes to development, adaptation, and flourishing in later life.

### 5.1 Contributions to human-AI interaction and active aging research

The first contribution concerns human-AI interaction research. Much of the human-AI interaction literature has examined relational concepts such as social presence, trust, empathy, and user expectations in technologically mediated interaction (Lee and See, 2004; Nass and Moon, 2000). These concepts explain why users often respond to conversational systems as if they were social actors. Yet they do not fully capture the developmental consequences of AI engagement. The capability perspective redirects attention from interaction quality to interaction outcomes. The issue is no longer whether conversational AI successfully imitates human relationships, but whether it expands individuals' capabilities and supports human flourishing.

The framework also extends active aging theory. Active aging scholarship has increasingly emphasized participation, autonomy, social engagement, and quality of life as multidimensional conditions of later-life development (Fernández-Ballesteros et al., 2013; Foster and Walker, 2015). Existing frameworks generally focus on health status, social support, economic resources, and environmental conditions. Conversational AI introduces a new category of developmental resource. As opportunities for participation, learning, healthcare access, and social engagement are increasingly mediated through digital infrastructures, technological environments become part of the ecology through which active aging is achieved. This perspective aligns with contemporary views of aging as adaptive rather than decline-centered (Baltes, 1997).

### 5.2 Digital inclusion, mental health, and ethical implications

The framework also reframes digital inclusion. Research on digital inequality has increasingly moved beyond access to examine differences in skills, usage patterns, and the benefits individuals derive from digital engagement (Gell et al., 2015; Helsper, 2012). Although these dimensions remain important, they provide only a partial explanation of meaningful participation. A capability perspective suggests that digital inclusion should be evaluated according to the opportunities individuals are able to realize through digital engagement. This distinction is particularly relevant for older adults, whose digital engagement is shaped by everyday ICT practices, perceived usefulness, social support, and opportunities to convert digital access into meaningful participation (Gell et al., 2015). Conversational AI may reduce some barriers by lowering technical demands and creating more intuitive pathways to participation.

The framework also contributes to discussions of conversational AI and mental health. Current digital mental health research increasingly situates chatbots within a broader ecosystem of apps, remote monitoring, social media, and virtual interventions, while emphasizing the need for stronger evidence, safety oversight, and clinical integration (Firth et al., 2017; Torous et al., 2021). These approaches have demonstrated promising outcomes, but symptom reduction represents only one dimension of psychological wellbeing. The capability perspective broadens evaluation by asking whether AI systems enhance adaptive capacities, including self-reflection, emotional regulation, social participation, and digital confidence.

Ethically, the capability perspective shifts evaluation from safety alone to agency. Responsible AI scholarship emphasizes transparency, accountability, fairness, privacy, and human oversight as central conditions for ethically acceptable AI systems, particularly in socially sensitive domains (Floridi et al., 2018; Jobin et al., 2019). Earlier assessments of smartphone-based conversational agents showed that such systems often responded inconsistently to questions involving mental health, interpersonal violence, and physical health, underscoring the need for safety-sensitive evaluation in vulnerable contexts (Miner et al., 2016). These concerns remain essential, especially for populations that may experience heightened vulnerability. Yet the critical issue is also whether AI expands or constrains individuals' opportunities for autonomous action. Conversational AI may increase independence by facilitating access to information and supporting decision-making, but excessive reliance may reduce opportunities for independent judgment, interpersonal interaction, or critical reflection. For older adults, responsible conversational AI should therefore be evaluated not only by technical safety or usability, but also by whether it preserves opportunities for independent judgment, meaningful human contact, and self-directed participation.

### 5.3 Future research agenda

The framework generates several priorities for future research. First, longitudinal studies are needed to determine whether sustained interaction with conversational AI contributes to enduring changes in cognitive, emotional, social, and digital capabilities. Second, future research should develop multidimensional measures capable of assessing capability formation rather than relying only on loneliness, usability, satisfaction, or technology acceptance. Third, research should examine how differences in health, education, socioeconomic resources, social support, digital experience, and individual goals shape the capability effects of conversational AI among older adults. Fourth, comparative studies are needed to explore how welfare systems, family structures, social norms, and digital infrastructures shape the relationship between AI and capability formation. Finally, future research should test conversational AI in real-world Ambient Assisted Living environments using longitudinal multimodal designs that integrate conversational, behavioral, and physiological data while ensuring privacy, reliability, and user autonomy (Johnson et al., 2026; Liu et al., 2026).

These contributions suggest that conversational AI should be understood as part of a broader developmental ecology rather than as a stand-alone technological intervention. The central issue is not whether AI can simulate companionship, but whether AI contributes to the expansion of capabilities that enable individuals to learn, adapt, participate, and flourish. As AI systems continue to evolve toward more adaptive and multimodal forms, future research should examine not only what these systems can provide, but also how older adults can meaningfully transform these technological opportunities into valued capabilities.

## 6 Conclusion

This paper has argued that the dominant digital companionship paradigm offers only a partial explanation of how conversational AI may influence older adults' psychological wellbeing. While this paradigm captures the value of emotional support, social presence, and loneliness reduction, it provides limited insight into the developmental processes through which AI interaction may support later-life adaptation and flourishing.

Drawing on the Capability Approach, this paper reconceptualizes conversational AI as a capability-building resource. It proposes that conversational AI contributes to wellbeing by expanding older adults' cognitive, emotional, social, and digital capabilities, rather than by merely substituting for human companionship. The AI-Augmented Active Aging Framework further explains how AI affordances create opportunities for capability formation, which in turn supports psychological wellbeing and active aging outcomes. The framework does not assume that conversational AI generates uniform benefits or that all older adults require companionship or capability support. Its effects depend on users' goals, existing strengths, social resources, digital experience, and the reliability and accessibility of the system. Supportive conversion conditions may facilitate capability formation, whereas adverse conditions may produce limited benefits, capability divergence, or capability erosion.

The central contribution of this paper lies in shifting attention from what conversational AI provides to what older adults become able to do through engagement with AI. As conversational AI becomes increasingly embedded in everyday life, its value should be assessed according to whether it expands or constrains older adults' opportunities for autonomous, connected, and self-determined living.
